# Establishing a trauma registry in Bhutan: needs and process

**DOI:** 10.1186/2193-1801-2-231

**Published:** 2013-05-20

**Authors:** Stephen C Morris, Nicolas Manice, Taylor Nelp, Tashi Tenzin

**Affiliations:** Emergency Medicine, University of Washington School of Medicine Seattle, 446 27th Ave East, Seattle, WA 98112 USA; Stony Brook School of Medicine, Stony Brook, NY USA; College of Physicians and Surgeons Columbia University, New York, NY USA; Neurosurgery and Emergency Services Director Jigme Dorji Wangchuck National Referral Hospital, Thimphu, Bhutan

**Keywords:** Bhutan, Trauma registry, Emergency health systems

## Abstract

**Background:**

Globally, trauma represents a growing and significant burden of disease. Many health systems have limited metrics with which to guide development and appropriately inform policy and management decisions with regard to trauma related health care delivery.

**Findings:**

This paper outlines the establishment of need for improved trauma related metrics in the country of Bhutan and the process of development of a trauma registry at Jigme Dorji Wangchuck National Referral Hospital to meet that need.

**Conclusions:**

Trauma registries are important tools allowing health systems to respond to the shifting burden of disease; successful establishment of a trauma registry requires an understanding of the health system and broad institutional support.

## Introduction

This project outlines the concepts and process involved in establishing a trauma registry at Jigme Dorji Wangchuck National Referral Hospital Thimphu, Bhutan (JDWNRH) as a model for the country of Bhutan. A need for improved emergency medical care was established by the Royal Government of Bhutan and collaborating partners and improved trauma related metrics were identified as critical to informed development.

Recent changes in the understanding of trauma related outcomes and demographics have led to a trend in international policy, funding and programmatic implementation emphasizing trauma care and injury prevention in the developing world (Laxminarayan et al. 2006; Mock et al. 1998; Mock et al. 2003). In particular, significant differences in trauma outcomes point to great potential for health system and trauma care development (Christensen et al. 2010). In an attempt to better understand and manage trauma care in Bhutan a collaboration was formed between the Royal Government of Bhutan, the Bhutan Foundation, Brigham and Women’s Hospital Department of Emergency Medicine and International Emergency Medicine Fellowship and Harvard Humanitarian Initiative to establish a trauma registry at JDWNRH.

## Background

The country of Bhutan is a land locked mountainous region of central Asia with a population of 700,000 and borders China and India. An overwhelmingly rural and agricultural society (estimated 85% for both) with limited health and economic infrastructure, Bhutan faces significant challenges with regard to health care delivery (Sustainable Development Department, Food and Agriculture Organization (FAO) of the United Nations 2999). The government provides western and traditional medical services care through 31 hospitals, 178 basic health unit clinics and 654 outreach clinics representing coverage of 90% of the population (Tobgay et al. 2011). Recent political changes, including transition from a monarchy to a democracy, and the opening of personal restrictions are fostering economic and social change, but are also likely influencing population health. The country suffers from a lack of qualified health care professionals with only 52 doctors and 545 nurses in the country in 2007. Markers of health in Bhutan demonstrate significant traumatic burden of disease Although some markers of disease burden remain high, for example: 14.4 road traffic deaths per 100,000 population annually (WHO) efforts by the Royal Government of Bhutan with regard to health have had been significant. Despite these challenges maternal mortality, as a marker of overall health care delivery and services, has improved from ~940 to ~650 to ~255 per 100,000 live births in 1990, 1995 and 2000 respectively (World Health Organization 2012) +.

Injuries and the burden of injuries on the health care system are increasing in Bhutan, resulting in the prioritization and need for increased data on the part of the MoH. Data from the Monthly Morbidity Report (MMR) presented in the 2009 Annual Health Bulletin shows total number of Injuries and Poisoning increasing from 19,117 in 2004 to 26,330 in 2008, and increase of 37.7%. In addition the number of deaths attributed to Injuries and Poisoning increased from 13 in 2004 to 30 in 2008, an increase of 130%. This data correlates with global studies of trends that show dramatically increasing rates of injuries and associated death and disability (Murray & Lopez 1997; Krug et al. 2000). The WHO’s Violence and Injury Prevention and Disability section’s world report on road traffic prevention predicts that road trafic accident (RTA) deaths will increase by 83% in low-income and middle income countries and RTAs globally will become the third largest burden of disease by 2020 (WHO 2004). With an understanding of these trends both from the MMR and inferred from global trends it is likely that the number of trauma cases presenting for care in Bhutan has and will increase, highlighting the need for improved data to better understand the circumstances of trauma within the country and role of emergency care to improve outcomes.

### International efforts concerning trauma data

Trauma registries provide governments, policymakers and public health specialists with data to guide their decision-making (Eastridge et al. 2010). Policies to reduce harm and avoid accidents only work if targeted for the right population, the right time, and the right setting. Trauma registries allow for stratification of injury data to help policymakers identify high-risk populations, locations, personal actions, and infrastructure defects (Moore & Clark 2008). As part of the broader goal of strengthening trauma systems globally emphasis on monitoring of outcomes and identifying avoidable causes of death has been endorsed by the global community (Mock et al. 2005).

Examples of the use of trauma related data in policy making from low and middle income countries provide support for the establishment of other data collection tools. Drunk-driving laws in particular, are a positive example of effective policy change and injury reduction. In Thailand the MoH has used data on alcohol, helmet use and speeding to promote effective legislation (Nakahara et al. 2005). In addition, traffic patterns (location, speed, demographics, urbanization) and driver use patterns (alcohol use, seatbelts, speeding) when correlated with trauma data lead to a paradigm shift in policy towards prevention (Suriyawongpaisal 2003). Through aggregate data collection, death rates from motor vehicle accidents in Columbia were shown to be occurring overwhelmingly (80%) in males and increasing as the economy of the country changed. This allowed policy makers to target interventions to specific populations and areas of rapid economic change (Posada et al. 2000). A meta-analysis of 16 studies of injury patterns related to motor vehicle accidents and policy interventions to slow traffic in congested areas showed reductions in the number of accidents (Bunn et al. 2003).

Details from trauma registries were used to change laws governing the legal age for purchase of alcohol, timing of alcohol sales, and penalties for drunk driving (Grube & Stewart 2004; McMillan & Lapham 2006). Combined policy efforts to reduce drunken driving deaths have been shown to greatly reduce the number of deaths in the United States (DeJong & Hingson 1998). A study from Brazil showed stepwise improvements in the number of traffic related deaths from seatbelt interventions, alcohol interventions and improvement in the overall traffic code (de Andrade et al. 2008).

### Role of a trauma registry

Health systems working to improve their trauma care and monitor patient outcomes have adopted trauma registries as a means of gathering and processing information (Cameron et al. 2005; Cameron et al. 2004). Health system analysis long defined by hospital based deaths has evolved with greater availability of data. Data on more socially relevant outcomes such as morbidity, hospital length of stay, and long term quality of life are becoming available to policy makers and managers with establishment of more complex databases (Mann et al. 1999).

Collection of trauma data is inherently fraught with difficulty including the necessity of clinical staff for data collection. Meta analysis of large trauma registries demonstrate under-reporting and systemic error (Wynn et al. 2001).

## Methods

### Trauma registry introduction

**Figure 1:**
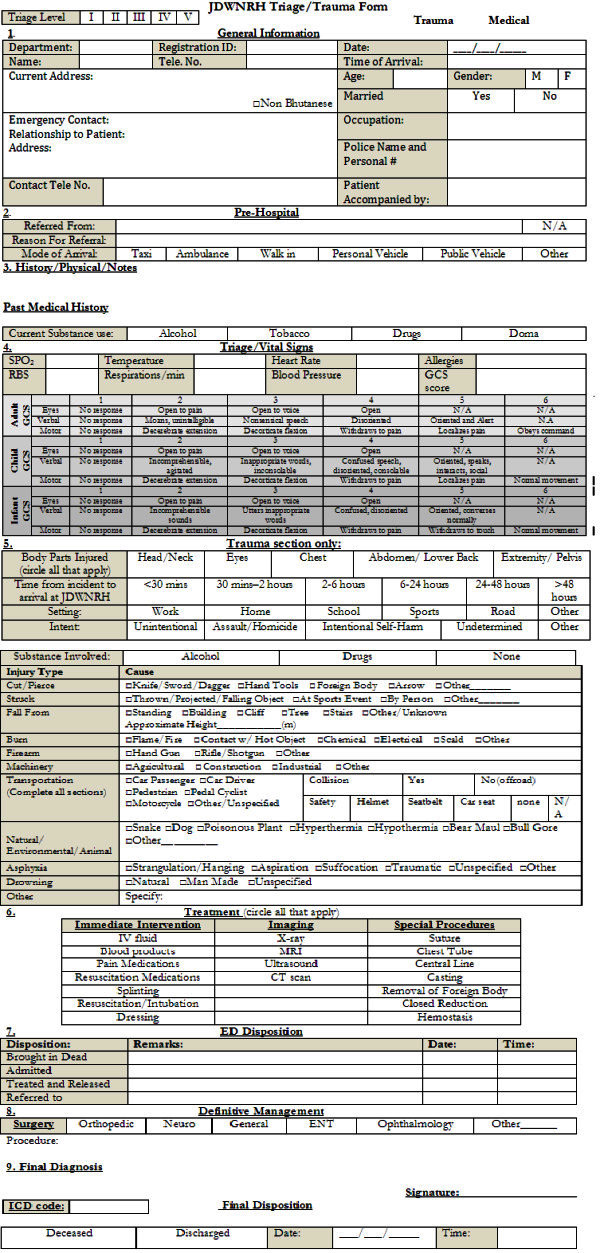
**JDWNRH trauma registry form.**

An initial assessment of trauma related data collection was conducted concurrent with an evaluation and needs assessment of overall emergency medicine services in 2009 (Rosborough et al. 2999). This involved evaluation of current trauma data collection methods and practices within the informal, quantitative and qualitative evaluations. Recommendations included specific data points to be collected for a trauma registry based on the evaluation and international models (Figure 1).

### Trauma registry logistics

At the behest of the MoH and coordinated by the Bhutan Foundation, two volunteer staff members were dispatched to the DJWNRH for six months beginning in the June of 2010. The goal of the assignment was to establish a model trauma registry at DJWNRH the country’s main referral hospital. The two staff members were briefed and supervised by DJWNRH and MoH, Harvard Humanitarian Initiative, staff from the Bhutan Foundation, and visiting American emergency medicine physicians working at DJWNRH through Health Volunteers Oversease (HVO).

While in Bhutan the two staff members helped build a core group of staff, the ‘trauma team’ focused on the development and implementation of the trauma registry. This group included management, clinical staff and administrators involved in hospital services related to trauma patients. Definitions were agreed upon and the data points were chosen based on addressing the important elements of demographics, prehospital care, vital signs representing clinical condition, mechanism or nature of injury, treatment and disposition. A trauma registry form was developed and piloted with subsequent implementation in September 2010.

### Trauma registry establishment and challenges

**Figure 2:**
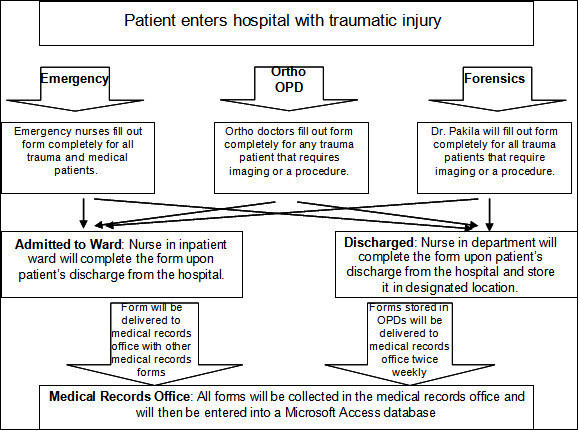
**Schematic of JDWNRH trauma registry processes.**

Establishment of the trauma registry at JDWNRH was met with some specific challenges. First, while there was interest in the benefits of the registry, as evidenced by MoH efforts and a previous attempt at establishing a registry several years earlier, clinical workload and commitment of resources hindered the process. Communication between the MoH and clinical staff with regard to data use and priority setting was not clear. Arrival of trauma patients occurred at several venues (ED, Orthopedics, Forensics, some transfers went straight to Surgery, ENT reported some trauma) requiring many staff to be trained and adding logistical difficulties. Three clinic departments – the emergency department, orthopedics, and forensics – represented the bulk of trauma patients and were trained to administer the registry. The data processing was conducted by the medical records office and staff of the trauma team with the goal of eventually transferring the data to the MoH for further analysis and use in policy and management decision making. Document flow through the hospital was a great challenge, requiring layers of shifting responsibility. (A copy of the flow diagram is presented in Figure 2). In addition, logistical challenges such as incomplete data on the form and lost forms hampered the pilot. Despite efforts to increase training and to fill responsibility gaps by the trauma team, these concerns represent complications to the future of the system.

### Trauma registry limitations

Many potential limitations existed within the establishment of the trauma registry at DJWNRH. Uncertain buy-in and variable long-term commitment on the part of the MoH and DJWNRH were exacerbated by the time required to implement the project. Use of on the ground staff (as opposed to technical experts) concerning policy implications of data points was also likely an important limitation. Implementation of the paper trauma registry before the planned implementation of the computerized medical record was necessary but less than ideal. Finally, given the limited number of clinical staff the untoward affect of using staff time to develop a tool of unclear clinical significance and longevity was of great concern.

## Conclusions

The project outlined above represented initial attempts to confront the unmet needs of the people of Bhutan and desire of the government to improve health outcomes with regards to trauma and emergency care. Many of the challenges facing Bhutan’s goals for improving emergency medical care are similar to those found in other resource poor settings: economic and logistical challenges to scaling up care, lack of trained health care personnel and limited training opportunities, and difficulty prioritizing interventions and health care systems investment. Improved metrics as a goal of this project, has potential to confront some of the limitations inherent within systems with limited resources. In addition, given Bhutan’s reliance on partners for development, the country’s ability to guide its development may be influenced by external priorities. Improved metrics may allow the MoH greater ability to influence development partners towards more impact driven interventions.
